# ﻿A new odorous frog species of *Odorrana* (Amphibia, Anura, Ranidae) from Guizhou Province, China

**DOI:** 10.3897/zookeys.1192.114315

**Published:** 2024-02-19

**Authors:** Shi-Ze Li, Ji-Jun Chen, Hai-Jun Su, Jing Liu, Xiu-Jun Tang, Bin Wang

**Affiliations:** 1 Department of Food Science and Engineering, Moutai Institute, Renhuai 564500, China Chengdu Institute of Biology, Chinese Academy of Sciences Chengdu China; 2 Chengdu Institute of Biology, Chinese Academy of Sciences, Chengdu 610041, China Moutai Institute Renhuai China; 3 Leigongshan National Nature Reserve Administration, Leishan 557100, China Leigongshan National Nature Reserve Administration Leishan China; 4 College of Forestry, Guizhou University, Guiyang 550025, China Guizhou University Guiyang China

**Keywords:** Leigong Mountain, molecular phylogenetic analysis, morphology, new species

## Abstract

The frog genus *Odorrana* is distributed across east and southeastern Asia. Based on morphological differences and molecular phylogenetics, a new species of the genus occurring from Leigong Mountain in Guizhou Province, China is described. Phylogenetic analyses based on DNA sequences of the mitochondrial *12S rRNA*, *16S rRNA*, and *ND2* genes supported the new species as an independent lineage. The uncorrected genetic distances between the *12S rRNA*, *16S rRNA*, and *ND2* genes between the new species and its closest congener were 5.0%, 4.9%, and 16.3%, respectively. The new species is distinguished from its congeners by a combination of the following characters: body size moderate (SVL 39.1–49.4 mm in males, 49.7 mm in female); head width larger than head length; tympanum distinctly visible; small rounded granules scattered all over dorsal body and limbs; dorsolateral folds absent; heels overlapping when thighs are positioned at right angles to the body; tibiotarsal articulation reaching the level between eye to nostril when leg stretched forward; vocal sacs absent in male and nuptial pads present on the base of finger I.

## ﻿Introduction

The odorous frogs of the genus *Odorrana* Fei, Ye & Huang, 1990 inhabit mountain streams at elevations of about 200–2000 m and can also be found on rocks or branches near the riverbed, ranging from Japan, southern China and Indochina, northeastern India, Myanmar and Thai-Malay Peninsula, Java, Sumatra, and Borneo (Frost 2024). Phylogenetic studies indicate that *Odorrana* is monophyletic (Chen et al. 2013). The genus currently consists of 65 species (Frost 2024), of which 42 occur in China and 27 species are endemic to China (Fei et al. 2012; Amphibia China 2024; Frost 2024).

Systematic arrangements in this genus have been controversial. Ye and Fei (2001) suggested four species groups (*O.andersonii*, *O.kuangwuensis*, *O.schmackeri*, and *O.livida* species groups) based on a morphological study. Fei et al. (2005) established two subgenera (*Odorrana* Fei, Ye & Huang, 1990 and *Bamburana* Fei, Ye, Huang, Jiang & Xie, 2005) within *Odorrana*. Molecular phylogenetic studies support the division of species groups within *Odorrana* but not the two subgenera (Che et al. 2007). Subsequently, Fei et al. (2009) divided the Chinese *Odorrana* species into six species groups (*O.tormota*, *O.taiwaniana*, *O.graminea*, *O.margaretae*, *O.schmackeri*, and *O.andersonii* species groups). These divisions have been accepted by some researchers (Pham et al. 2016a, b; Li et al. 2018a) but others have rejected the monophyly of the *O.margaretae*, *O.schmackeri*, and *O.andersonii* species groups (Chen et al. 2013). The species diversity in the genus is also indicated as underestimated in these phylogenetic frameworks.

Guizhou Province is one of the areas of the most abundant amphibians in China, and in the last five years a series of new frog species have been described from this region (Frost 2024; Amphibia China 2024). During fieldwork in Leigongshan Nature Reserve, Leishan County, Guizhou Province, China, between March to October 2023, seven *Odorrana* specimens were collected. Morphologically, these specimens most closely *O.huanggangensis* Chen, Zhou & Zheng, 2010, and *O.wuchuanensis* (Xu, 1983), but differs from these two species by the presence of small, rounded granules scattered all over the dorsal body and limbs, and the vocal sacs are absent in the male. To further distinguish these specimens, we conducted phylogenetic analyses based on mitochondrial DNA and morphological comparisons. All analyses consistently indicated that the specimens from Leigongshan Nature Reserve are a new taxon, described herein as a new species.

## ﻿Materials and methods

### ﻿Sampling

**Figure 1.**
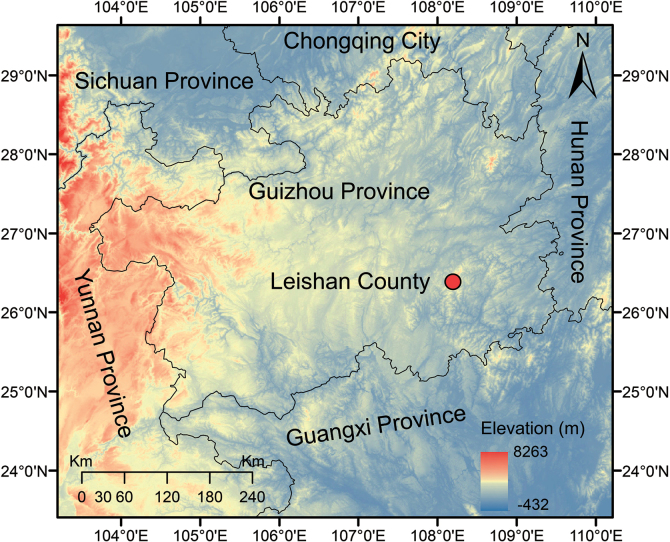
Geographical location of the type locality of *Odorranaleishanensis* sp. nov., Leigongshan Nature Reserve, Leishan County, Guizhou Province, China.

Seven specimens (♀ *n* = 1; ♂ *n* = 6) of the unnamed taxon were collected by hand from Leigongshan Nature Reserve, Guizhou Province, China (Fig. 1) and the field work was approved by the Management Office of the Leigongshan Nature Reserve (project number: P5226002023000019). The Animal Care and Use Committee of Guizhou University provided full approval for this research (Number: EAE-GZU-2022-T115). All specimens were fixed in 10% buffered formalin for 10 h, and then later transferred to 75% ethanol. Tissue samples were preserved separately in 95% prior to fixation. Specimens collected in this work were deposited in Moutai Institute (**MT**), Guizhou Province, China. In addition, 12 tissue samples containing two *Odorranafengkaiensis* Wang, Lau, Yang, Chen, Liu, Pang & Liu, 2015, one *O.hainanensis* Fei, Ye & Li, 2001, one *O.bacboensis* (Bain, Lathrop, Murphy, Orlov & Ho, 2003), three *O.ichangensis* Chen, 2020, and two *O.hejiangensis* (Deng & Yu, 1992) were used (Table 1).

**Table 1.** Information of samples used in molecular phylogenetic analyses in this study; a slash (/) indicates information absent.

ID	Species	Locality	Voucher number	GenBank accession number			Citation
				12s	16s	ND2	
1	*Odorranaleishanensis* sp. nov.	Leigongshan Nature Reserve, Leishan, Guizhou, China	MT LS20230806010	OR879770	OR879754	OR863727	this study
2	*Odorranaleishanensis* sp. nov.	Leigongshan Nature Reserve, Leishan, Guizhou, China	MT LS20230805001	OR879771	OR879755	OR863728	this study
3	*Odorranaleishanensis* sp. nov.	Leigongshan Nature Reserve, Leishan, Guizhou, China	MT LS20230811024	OR879772	OR879756	OR863729	this study
4	*Odorranaleishanensis* sp. nov.	Leigongshan Nature Reserve, Leishan, Guizhou, China	MT LS20230729013	OR879773	OR879757	OR863730	this study
5	*Odorranaleishanensis* sp. nov.	Leigongshan Nature Reserve, Leishan, Guizhou, China	MT LS20230806018	OR879774	OR879758	OR863731	this study
6	*Odorranaleishanensis* sp. nov.	Leigongshan Nature Reserve, Leishan, Guizhou, China	MT LS20230711020	OR879775	OR879759	OR863732	this study
7	*Odorranaleishanensis* sp. nov.	Leigongshan Nature Reserve, Leishan, Guizhou, China	MT LS20230717001	OR879776	OR879760	OR863733	this study
8	*Odorranafengkaiensis*	Heishiding Nature Reserve, Fengkai, Guangdong, China	SYS a002262	KT315354	KT315375	OR863743	Wang et al. 2015; this study
9	*Odorranafengkaiensis*	Heishiding Nature Reserve, Fengkai, Guangdong, China	SYS a002263	KT315355	KT315376	OR863744	Wang et al. 2015; this study
10	*Odorranafengkaiensis*	Heishiding Nature Reserve, Fengkai, Guangdong, China	SYS a002273	KT315356	KT315377	/	Wang et al. 2015
11	*Odorranahainanensis*	Wuzhishan city, Hainan, China	HNNU0606105	KF184996	KF185032	/	Wang et al. 2015
12	*Odorranahainanensis*	Diaoluoshan Forest Park, Lingshui, Hainan, China	SYS a002260	KT315362	KT315383	OR863741	this study
13	*Odorranabacboensis*	Bainan village, Napo, Guangxi, China	SYS a001046	KT315364	KT315385	OR863742	this study
14	*Odorranabacboensis*	Khe Moi, Nghe An,Vietnam	ROM 13044	AF206099	DQ650569	/	Chen et al. 2005
15	*Odorranabacboensis*	Hekou, Yunnan, China	HNNU HK001	KF185008	KF185044	/	Chen et al. 2013
16	*Odorranaschmackeri*	Songtao, Guizhou, China	MT ST20210622001	OR879782	OR879768	OR863745	this study
17	*Odorranaschmackeri*	Yichang City, Hubei, China	HNNU0908II349	KF185011	KF185047	/	Chen et al. 2013
18	*Odorranaschmackeri*	Songtao, Guizhou, China	MT ST20210622002	OR879782	OR879769	OR863746	this study
19	*Odorranakweichowensis*	Lengshuihe Nature Reserve, Jinsha, Guizhou, China	CIBjs20150803008	MH193538	MH193552	MH193606	Li et al. 2018
20	*Odorranakweichowensis*	Lengshuihe Nature Reserve, Jinsha, Guizhou, China	CIBjs20171014001	MH193539	MH193551	MH193605	Li et al. 2018
21	*Odorranasangzhiensis*	Sangzhi, Hunan, China	CSUFT 4308220046	MW465705	MW464864	/	Zhang et al. 2021
22	*Odorranasangzhiensis*	Sangzhi, Hunan, China	CSUFT 4308220051	MW465701	MW464865	/	Zhang et al. 2021
23	*Odorranasangzhiensis*	Sangzhi, Hunan, China	CSUFT 4308220048	MW465702	MW464861	/	Zhang et al. 2021
24	*Odorranaichangensis*	Zhijin, Guizhou, China	MT ZJ20210814003	/	OR879766	OR863739	this study
25	*Odorranaichangensis*	Zhijin, Guizhou, China	MT ZJ20210814004	/	OR879767	OR863740	this study
26	*Odorranaichangensis*	Yichang City, Hubei, China	SYS a005475	OR879781	OR879765	OR863738	this study
27	*Odorranahejiangensis*	Chishui, Guizhou, China	MT CS20200605007	OR879779	OR879763	OR863736	this study
28	*Odorranahejiangensis*	Chishui, Guizhou, China	MT CS20200605008	OR879780	OR879764	OR863737	this study
29	*Odorranahejiangensis*	Hejiang, Sichuan, China	HNNU1007I202	KF185016	KF185052	/	Chen et al. 2013
30	*Odorranatianmuii*	Lin’an, Zhejiang, China	HNNU707071	KF185004	KF185040	/	Chen et al. 2013
31	*Odorranatianmuii*	Lin’an, Zhejiang, China	SYS a002680	OR879777	OR879761	OR863734	this study
32	*Odorranatianmuii*	Lin’an, Zhejiang, China	SYS a002681	OR879778	OR879762	OR863735	this study
33	*Odorranahuanggangensis*	Fanjingshan Nature Reserve, Jiangkou, Guizhou, China	CIBFJS20150502002	MH193532	MH193565	MH193614	Li et al. 2018
34	*Odorranahuanggangensis*	Leigongshan Nature Reserve, Leishan, Guizhou, China	CIBLS20140818005	MH193530	MH193564	MH193612	Li et al. 2018
35	*Odorranahuanggangensis*	Wuyishan Nature Reserve, Fujian, China	HNNU0607001	KF185023	KF185059	/	Chen et al. 2013
36	*Odorranaversabilis*	Leigongshan Nature Reserve, Leishan, Guizhou, China	HNNU003	KF185019	KF185055	/	Chen et al. 2013
37	*Odorrananasuta*	Wuzhishan, Hainan, China	HNNU051119	KF185017	KF185053	/	Chen et al. 2013
38	*Odorranaexiliversabilis*	Wuyishan, Fujian, China	HNNU0607032	KF185020	KF185056	/	Chen et al. 2013
39	*Odorranayentuensis*	Guangxi, China	NHMG1401035	MH665669	MH665675	/	Mo et al. 2015
40	*Odorrananasica*	HaTinh, Vietnam	AMNH A161169	DQ283345	DQ283345	/	Frost et al. 2006
41	*Odorranatormota*	Huangshan, Anhui, China	AM04005	DQ835616	DQ835616	DQ835616	Su et al. 2007
42	*Odorrananarina*	Okinawa Island, Japan	/	AB511287	AB511287	AB600990	Kurabayashi et al. 2010
43	*Odorranaamamiensis*	Tokunoshima, Ryukyu, Japan	KUHE:24635	AB200923	AB200947	AB600991	Matsui et al. 2006
44	*Odorranasupranarina*	Iriomotejima, Ryukyu	KUHE:12898	AB200926	AB200950	/	Matsui et al. 2006
45	*Odorranaswinhoana*	Nantou, Taiwan, China	HNNUTW9	KF185010	KF185046	/	Chen et al. 2013
46	*Odorranautsunomiyaorum*	Iriomotejima, Ryukyu	KUHE:12896	AB200928	AB200952	/	Matsui et al. 2006
47	*Odorranahosii*	Kuala Lumpur, Malaysia	IABHU 21004	AB511284	AB511284	/	Kurabayashi et al. 2010
48	*Odorranagraminea*	Wuzhishan, Hainan, China	HNNU0606123	KF185002	KF185038	/	Chen et al. 2013
49	*Odorranachloronota*	Ha Giang, Vietnam	AMNH A163935	DQ283394	DQ283394	/	Frost et al. 2006
50	*Odorranalivida*	Prachuap Kirikhan, Thailand	FMNH 263415	KF771294	DQ650613	DQ650546	Stuart et al. 2006b
51	*Odorranaleporipes*	Shaoguan, Guangdong, China	HNNU1008I099	KF185000	KF185036	/	Chen et al. 2013
52	*Odorranaaureola*	Phu Rua, Loei, Thailand	FMNH 265919	/	DQ650564	DQ650500	Stuart et al. 2006
53	*Odorranamorafkai*	Tram Lap, Vietnam	ROM 7446	AF206103	AF206484	/	Chen et al. 2005
54	*Odorranabanaorum*	Tram Lap, Vietnam	ROM 7472	AF206106	AF206487	/	Chen et al. 2005
55	*Odorranajunlianensis*	Junlian, Sichuan, China	HNNU002JL	KF185022	KF185058	/	Chen et al. 2013
56	*Odorranagrahami*	Kunming, Yunnan, China	HNNU1008II016	KF185015	KF185051	/	Chen et al. 2013
57	*Odorranahmongorum*	Lao Cai, Vietnam	ROM 38605	/	EU861556	EU861585	Bain et al. 2009
58	*Odorranadaorum*	Sa Pa, Vietnam	ROM 19053	AF206101	AF206482	/	Chen et al. 2005
59	*Odorranaandersonii*	Longchuan County, Yunnan, China	HNNU001YN	KF185021	KF185057	/	Chen et al. 2013
60	*Odorranajingdongensis*	Jingdong, Yunnan, China	20070711017	KF185014	KF185050	/	Chen et al. 2013
61	*Odorranamargaretae*	Mt. Emei, Sichuan, China	HNNU20050032	KF184999	KF185035	/	Chen et al. 2013
62	*Odorranakuangwuensis*	Nanjiang, Sichuan, China	HNNU0908II185	KF184998	KF185034	/	Chen et al. 2013
63	*Odorranadulongensis*	Dulongjiang, Yunnan, China	KIZ035027	/	MW128102	/	Liu et al. 2021
64	*Odorranawuchuanensis*	Maolan National Nature Reserve, Libo County, Guizhou, China	GZNU20180608018	MW481342	MW481353	MW481364	Luo et al. 2021
65	*Odorranawuchuanensis*	Wuchuan, Guizhou Prov., China	HNNU019L	KF185007	KF185043	/	Chen et al. 2013
66	*Odorranamutschmanni*	Cao Bang, Vietnam	IEBR 3725	KU356762	KU356766	/	Pham et al. 2016b
67	*Odorranayizhangensis*	Nanling Nature Reserve, Ruyuan County, Guangdong, China	CIBHN201108149	MH193540	MH193560	MH193615	Li et al. 2018
68	*Odorranayizhangensis*	Yizhang, Hunan	HNNU1008I075	KF185012	KF185048	/	Chen et al. 2013
69	*Odorranalungshengensis*	Longsheng, Guangxi	HNNU70028	KF185018	KF185054	/	Chen et al. 2013
70	*Odorranalungshengensis*	Leigongshan Nature Reserve, Leishan, Guizhou, China.	CIBLS20140616006	MH193534	MH193554	MH193608	Li et al. 2018
71	*Odorranaanlungensis*	Anlong, Guizhou, China	HNNU1008I109	KF185013	KF185049	/	Chen et al. 2013
72	*Odorranachapaensis*	Lai Chau, Vietnam	AMNH A161439	DQ283372	DQ283372	/	Frost et al. 2006
73	*Odorranageminata*	Ha Giang, Vietnam	AMNH 163782	/	EU861546	EU861572	Bain et al. 2009
74	*Odorranaishikawae*	Amami Island, Japan	IABHU 5275	AB511282	AB511282	AB511282	Kurabayashi et al. 2010
75	*Odorranaabsita*	Xe Kong, Laos	FMNH 258107	/	EU861542	EU861568	Bain et al. 2009
76	*Odorranaliboensis*	Maolan National Nature Reserve, Libo, Guizhou, China	GZNU20180608007	MW481339	MW481350	/	Luo et al. 2021
77	*Odorranalipuensis*	Lipu, Guangxi, China	NHMG1303018	MH665670	MH665676	/	Mo et al. 2015
78	*Odorranaconcelata*	Longlinchang Village, Qingyuan, Guangdong,China	GEP a050	OP137167	OP137161	/	Lin et al. 2022
79	*Babinaadenopleura*	/	A-A-WZ001	NC_018771	NC_018771	NC_018771	Yu et al. 2012
80	*Nidiranadaunchina*	Emeishan, Sichuan, China	HNNU20060103	KF185029	KF185065	/	Chen et al. 2013
81	*Amolopsloloensis*	Shimian, Sichuan, China	SM-ZDTW-01	NC_029250	NC_029250	NC_029250	Xue et al. 2016
82	*Amolopsricketti*	Wugongshan, Jiangxi, China	AM13988	NC_023949	NC_023949	NC_023949	Li et al. 2016
83	*Glandiranatientaiensis*	Huangshan, Anhui, China	SCUM0405192CJ	KX269222	KX269222	KX269435	Yuan et al. 2016
84	*Sylviranaguentheri*	Fuzhou City, Fujian, China	SCUM-H002CJ	KX269219	KX269219	/	Yuan et al. 2016
85	*Pelophylaxnigromaculata*	Hongya, Sichuan, China	SCUM045199CJ	KX269216	KX269216	KX269431	Yuan et al. 2016
86	*Ranaweiningensis*	Weining County, Guizhou, China	SCUM0405171	KX269217	KX269217	KX269432	Yuan et al. 2016

### ﻿Molecular data and phylogenetic analyses

DNA was extracted from muscle tissue using a DNA extraction kit from Tiangen Biotech Co., Ltd. (Beijing). All samples were sequenced for three mitochondrial genes, partial 12S ribosomal RNA gene (*12S rRNA*), 16S ribosomal RNA gene (*16S rRNA*), and NADH dehydrogenase subunit 2 (*ND2*). The primers used for *12S rRNA* were P1 (5’- CCAGGCTTTACACTTTATGC -3’) and P2 (5’- GCGATTAAGTTGGGTAACGC -3’) following Kocher et al. (1989); for *12S rRNA* were P7 (5’- CGCCTGTTTACCAAAAACAT -3’) and P8 (5’- CCGGTCTGAACTCAGATCACGT’) following Simon et al. (1994); and for *ND2* were Gln-LND2 (5’-CCCTTTGCACTTCCTTTATGC-3’) and Ala-HND2 (5’-GGCCTGAGTTGCATTCATG-3’) following Li et al. (2015). PCR amplification reactions were performed in a 30 μl volume contains 1× High-Fidelity Master Mix (Chengdu TSINGKE Biological Technology Co. Ltd.) 15 μl, ddH_2_O 10 μl, 0.5 μM Forward primer 2 μl, 0.5 μM Revers primer 2 μl and 4.25 μg/μl DNA 1 μl, reaction with the following cycling conditions: an initial denaturing step at 95 °C for 4 min; 36 cycles of denaturing at 95 °C for 40 s, annealing at 47 °C (for ND2)/57 °C (for 12S and 16S) for 40 s and extending at 72 °C for 70 s, and a final extending step of 72 °C for 10 min. PCR products were purified with spin columns and then were sequenced with both forward and reverse primers, same as for the PCR. Sequencing was conducted using an ABI Prism 3730 automated DNA sequencer in Chengdu TSINGKE Biological Technology Co. Ltd. (Chengdu, China). All sequences were deposited in GenBank (see Table 1 for GenBank accession numbers). For phylogenetic analyses and genetic divergence analyses, we downloaded available corresponding sequence data for all related species from GenBank according to previous studies (Chen et al 2013; Li et al 2018a; for GenBank accession no. refer to Table 1).

86 Sequences were assembled and aligned using the Clustalw module in BioEdit v. 7.0.9.0 (Hall 1999) with default settings. The datasets were checked by eye and revised manually if necessary. Based on the *12S rRNA*, *16S rRNA*, *ND2*, and *12S rRNA* +*16S rRNA* + *ND2* concatenated dataset, phylogenetic analyses were conducted using maximum likelihood (ML) and Bayesian inference (BI) methods, implemented in PhyML 3.0 (Guindon et al. 2010) and MrBayes 3.12 (Ronquist and Huelsenbeck 2003), respectively, and the best-fit model was obtained by the Bayesian inference criteria (BIC) computed with PartitionFinder 2 (Lanfear et al. 2012). The analysis suggested that the best partition scheme was *12S rRNA*/*16S rRNA*/*ND2* genes. We selected GTR+R as the best model for *12S rRNA* and *16S rRNA* and the TN93 + I + G as the best model for the ND2 gene. For ML analyses conducted in PhyML 3.0, the bootstrap consensus tree inferred from 1000 replicates was used to estimate nodal supports of inferred relationships on phylogenetic trees. For Bayesian analyses conducted in MrBayes 3.12, four Markov chains were run for 50 million generations with sampling every 1000 generations until the trees reach convergence (split frequency < 0.05). The first 25% of trees were removed as the “burn-in” stage followed by calculation of Bayesian posterior probabilities and the 50% majority-rule consensus of the post burn-in trees sampled at stationarity. Finally, uncorrected *p*-distances (1000 replicates) between species based on *12S rRNA* (45 species), *16S rRNA* (51 species), and *ND2* (23 species) were calculated in MEGA 6.06 (Tamura et al. 2013).

### ﻿Morphological comparisons

Morphological measurements were made with dial calipers to nearest 0.1 mm (Wenzhou Weidu Electronics Co. Ltd., China). Twenty morphometric characters of 76 adults specimens were measured containing seven specimens of the undescribed taxon, 15 *Odorranahejiangensis*, eight *O.huanggangensis*, 13 *O.ichangensis*, nine *O.kweichowensis* Li, Xu, Lv, Jiang, Wei & Wang, 2018, ten *O.schmackeri* (Boettger, 1892), and 14 *O.wuchuanensis* following Fei et al. (2009) and Li et al. (2018a), abbreviated as follows:

**ED** eye diameter (distance from the anterior corner to the posterior corner of the eye);

**FL** foot length (distance from tarsus to the tip of fourth toe);

**HDL** head length (distance from the tip of the snout to the articulation of jaw);

**HDW** maximum head width (greatest width between the left and right articulations of jaw);

**HLL** hindlimb length (maximum length from the vent to the distal tip of the Toe IV);

**IND** internasal distance (minimum distance between the inner margins of the external nares);

**IOD** interorbital distance (minimum distance between the inner edges of the upper eyelids);

**LAL** length of lower arm and hand (distance from the elbow to the distal end of the Finger IV);

**ML** manus length (distance from tip of third digit to proximal edge of inner palmar tubercle);

**NED** nasal to eye distance (distance between the nasal and the anterior corner of the eye);

**NSD** nasal to snout distance (distance between the nasal the posterior edge of the vent);

**LW** lower arm width (maximum width of the lower arm);

**SVL** snout-vent length (distance from the tip of the snout to the posterior edge of the vent);

**SL** snout length (distance from the tip of the snout to the anterior corner of the eye);

**TFL** length of foot and tarsus (distance from the tibiotarsal articulation to the distal end of the Toe IV);

**THL** thigh length (distance from vent to knee);

**TL** tibia length (distance from knee to tarsus);

**TW** maximal tibia width;

**TYD** maximal tympanum diameter;

**UEW** upper eyelid width (greatest width of the upper eyelid margins measured perpendicular to the anterior-posterior axis).

To reduce the impact of allometry, a size-corrected value from the ratio of each character to SVL was calculated for the following morphometric analyses. Principal component analysis (PCA) of size-corrected variables and simple bivariate scatterplots was used to explore and reflect the morphometric differences between the undescribed taxon and the phylogenetic relationships closely and sympatric species contains *Odorranahejiangensis*, *O.huanggangensis*, *O.ichangensis*, *O.kweichowensis*, *O.schmackeri*, and *O.wuchuanensis*. One-way analysis of variance (ANOVA) was used to test the significance of differences on morphometric characters between the undescribed taxon and *O.hejiangensis*, *O.huanggangensis*, *O.ichangensis*, *O.kweichowensis*, *O.schmackeri*, and *O.wuchuanensis* in the males. The statistical analyses were performed using SPSS 21.0 (SPSS, Inc., Chicago. IL, USA), and differences were considered to be significant at *p* < 0.05.

Sex was determined by direct observation of calling behavior and the presence of internal vocal sac openings for males, as well as the presence of eggs on the abdomen for females. The presence or absence of nuptial pads/spines was examined by optical microscopy.

We compared the morphological characters of the undescribed taxon with other species of *Odorrana*. Comparative data were obtained from the literature for 65 species of *Odorrana* (all of the authorities of the 65 species were shown in Table 2). For comparison, we examined the type and/or topotype materials for *O.hejiangensis*, *O.huanggangensis*, *O.ichangensis*, *O.kweichowensis*, *O.schmackeri*, and *O.wuchuanensis* (Suppl. material 1).

**Table 2.** References for morphological characters for congeners of the genus *Odorrana*.

ID	*Odorrana* species	Citation
1	*O.absita* (Stuart & Chan-ard, 2005)	Stuart and Chan-ard 2005
2	*O.amamiensis* (Matsui, 1994)	Matsui 1994
3	*O.andersonii* (Boulenger, 1882)	Boulenger 1882
4	*O.anlungensis* (Liu & Hu, 1973)	Hu et al. 1973
5	*O.arunachalensis* Saikia, Sinha & Kharkongor, 2017	Saikia et al. 2017
6	*O.aureola* Stuart, Chuaynkern, Chan-ard & Inger, 2006	Stuart et al. 2006a
7	*O.bacboensis* (Bain, Lathrop, Murphy, Orlov & Ho, 2003)	Bain et al. 2003; Wang et al. 2015
8	*O.banaorum* (Bain, Lathrop, Murphy, Orlov & Ho, 2003)	Bain et al. 2003
9	*O.bolavensis* (Stuart & Bain, 2005)	Stuart and Bain 2005
10	*O.cangyuanensis* (Yang, 2008)	Yang 2008
11	*O.chapaensis* (Bourret, 1937)	Bourret 1937
12	*O.chloronota* (Günther, 1876)	Günther 1876; Che et al. 2020
13	*O.concelata* Wang, Zeng & Lin, 2022	Lin et al. 2022
14	*O.confusa* Song, Zhang, Qi, Lyu, Zeng & Wang, 2023	Song et al. 2023
15	*O.damingshanensis* Chen, Mo, Lin & Qin, 2024	Chen et al. 2024
16	*O.dulongensis* Liu, Che & Yuan, 2021	Liu et al. 2021
17	*O.exiliversabilis* Li, Ye & Fei, 2001	Fei et al. 2001b
18	*O.fengkaiensis* Wang, Lau, Yang, Chen, Liu, Pang & Liu, 2015	Wang et al. 2015
19	*O.geminata* Bain, Stuart, Nguyen, Che & Rao, 2009	Bain et al. 2009
20	*O.gigatympana* (Orlov, Ananjeva & Ho, 2006)	Orlov et al. 2006
21	*O.grahami* (Boulenger, 1917)	Boulenger 1917
22	*O.graminea* (Boulenger, 1900)	Boulenger 1900
23	*O.hainanensis* Fei, Ye & Li, 2001	Fei et al. 2001a
24	*O.heatwolei* (Stuart & Bain, 2005)	Stuart and Bain 2005
25	*O.hosii* (Boulenger, 1891)	Boulenger 1891
26	*O.hejiangensis* (Deng & Yu, 1992)	Deng and Yu 1992
27	*O.huanggangensis* Chen, Zhou & Zheng, 2010	Chen et al. 2010a
28	*O.ichangensis* Chen, 2020	Shen et al. 2020
29	*O.ishikawae* (Stejneger, 1901)	Stejneger 1901
30	*O.indeprensa* (Bain & Stuart, 2006)	Bain and Stuart 2006
31	*O.jingdongensis* Fei, Ye & Li, 2001	Fei et al. 2001a
32	*O.junlianensis* Huang, Fei & Ye, 2001	Ye and Fei 2001
33	*O.khalam* (Stuart, Orlov & Chan-ard, 2005)	Stuart and Chan-ard 2005
34	*O.kuangwuensis* (Liu & Hu, 1966)	Hu et al. 1966
35	*O.kweichowensis* Li, Xu, Lv, Jiang, Wei & Wang, 2018	Li et al. 2018
36	*O.livida* (Blyth, 1856)	Blyth 1856
37	*O.liboensis* Luo, Wang, Xiao, Wang & Zhou, 2021	Luo et al. 2021
38	*O.lipuensis* Mo, Chen, Wu, Zhang & Zhou, 2015	Mo et al. 2015; Pham et al. 2016a
39	*O.leporipes* (Werner, 1930)	Werner 1930
40	*O.lungshengensis* (Liu & Hu, 1962)	Liu and Hu 1962
41	*O.macrotympana* (Yang, 2008)	Yang 2008
42	*O.margaretae* (Liu, 1950)	Liu 1950
43	*O.mawphlangensis* (Pillai & Chanda, 1977)	Pillai and Chanda 1977; Mahony 2008
44	*O.mutschmanni* Pham, Nguyen, Le, Bonkowski & Ziegler, 2016	Pham et al. 2016a
45	*O.monjerai* (Matsui & Jaafar, 2006)	Matsui and Jaafar 2006
46	*O.morafkai* (Bain, Lathrop, Murphy, Orlov & Ho, 2003)	Bain et al. 2003
47	*O.nasica* (Boulenger, 1903)	Boulenger 1903
48	*O.nasuta* Li, Ye & Fei, 2001	Fei et al. 2001b
49	*O.narina* (Stejneger, 1901)	Stejneger 1901
50	*O.nanjiangensis* Fei, Ye, Xie & Jiang, 2007	Fei et al. 2007a
51	*O.orba* (Stuart & Bain, 2005)	Stuart and Bain 2005
52	*O.sangzhiensis* Zhang, Li, Hu & Yang, 2021	Zhang et al. 2021
53	*O.schmackeri* (Boettger, 1892)	Boettger (1892); Shen et al. (2020)
54	*O.sinica* (Ahl, 1927)	Ahl 1927 “1925”; Bain et al. 2003
55	*O.swinhoana* (Boulenger, 1903)	Boulenger 1903
56	*O.supranarina* (Matsui, 1994)	Matsui 1994
57	*O.splendida* Kuramoto, Satou, Oumi, Kurabayashi & Sumida, 2011	Kuramoto et al. 2011
58	*O.tianmuii* Chen, Zhou & Zheng, 2010	Chen et al. 2010b
59	*O.tiannanensis* (Yang & Li, 1980)	Yang and Li 1980
60	*O.tormota* (Wu, 1977)	Wu 1977
61	*O.utsunomiyaorum* (Matsui, 1994)	Matsui 1994
62	*O.versabilis* (Liu & Hu, 1962)	Liu and Hu 1962
63	*O.wuchuanensis* (Xu, 1983)	Wu et al. 1983
64	*O.yentuensis* Tran, Orlov & Nguyen, 2008	Tran et al. 2008; Lu et al. 2016
65	*O.yizhangensis* Fei, Ye & Jiang, 2007	Fei et al. 2007b

## ﻿Results

### ﻿Phylogenetic analyses

**Figure 2.**
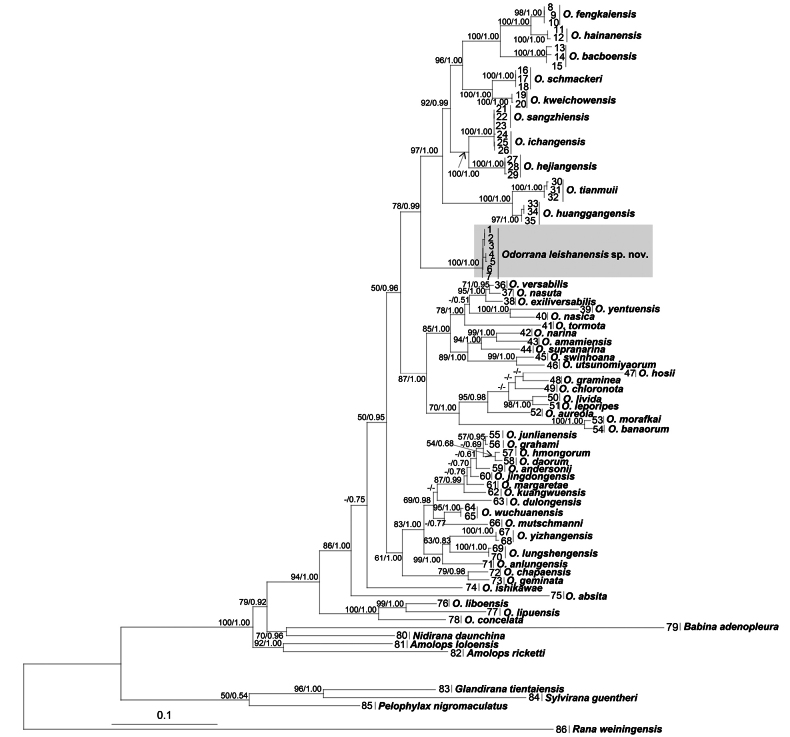
Maximum likelihood (ML) tree of the genus *Odorrana* reconstructed based on the *12S rRNA*, *16S rRNA*, and *ND2* gene sequences. ML bootstrap supports (BS)/Bayesian posterior probability (BPP) are denoted beside each node, and “-” denotes BS < 50% or BPP < 0.60. Samples 1–86 refer to those listed in Table 1.

The ML and BI phylogenetic trees were constructed based on *12S rRNA* (400 bp), *16S rRNA* (484 bp), *ND2* (915 bp), and *12S rRNA* +*16S rRNA* + *ND2* concatenated dataset. Both the independent dataset and concatenated dataset of ML and BI analyses resulted in essentially identical topologies with high node supporting values. The specimens of the undescribed taxon were clustered into an independent clade, sharing a sister relationship with the clade containing *Odorranaschmackeri*, *O.kweichowensis*, *O.fengkaiensis*, *O.hainanensis*, *O.bacboensis*, *O.ichangensis*, *O.hejiangensis*, *O.tianmuii* Chen, Zhou & Zheng, 2010, and *O.huanggangensis* with high node support values (0.99 in BI and 78% in ML, Fig. 2; 0.98 in BI and 92% in ML, Suppl. material 5; 0. 80 in BI and 50% in ML, Suppl. material 6; 0.99 in BI and 70% in ML, Suppl. material 7).

The mean genetic distance between the undescribed taxon and its closely related species is 5.0%, 4.9%, and 16.3% on 12S, 16S, and ND2, respectively, much higher than that between many pairs of species in the genus *Odorrana* (Suppl. materials 2–4).

### ﻿Morphological analyses

**Figure 3.**
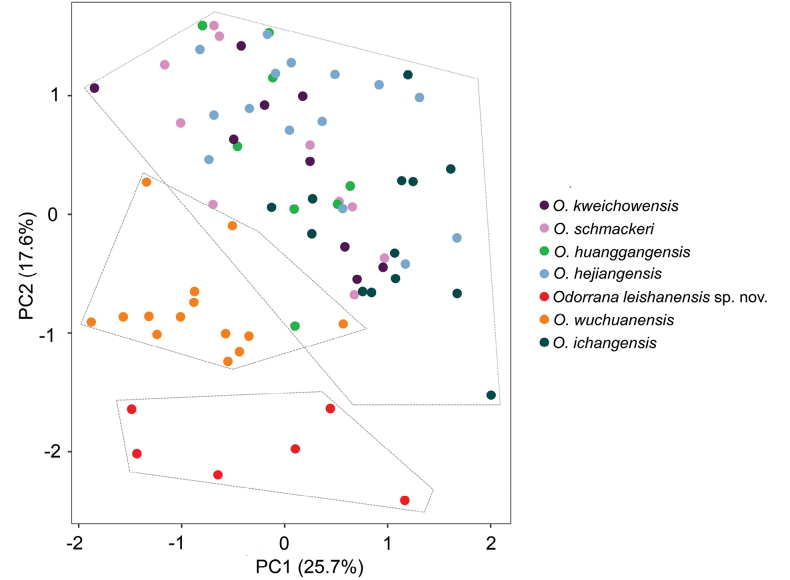
Plots of the first principal component (PC1) versus the second (PC 2) for *Odorranaleishanensis* sp. nov., *O.hejiangensis*, *O.huanggangensis*, *O.ichangensis*, *O.kweichowensis*, *O.schmackeri*, and *O.wuchuanensis* in males from a principal component analysis.

The results of ANOVA indicated that in male, the undescribed taxon was significantly different from *Odorranahejiangensis*, *O.huanggangensis*, *O.ichangensis*, *O.kweichowensis*, *O.schmackeri*, and *O.wuchuanensis* in many morphometric characters (all P values < 0.05; Table 3). In PCA for males, the total variation of the first two principal components was 43.3%, on the two-dimensional plots of PC1 vs PC2, the undescribed taxon could be separated from *O.hejiangensis*, *O.huanggangensis*, *O.ichangensis*, *O.kweichowensis*, *O.schmackeri*, and *O.wuchuanensis* (Fig. 3). Detailed morphological comparisons revealed discrete diagnostic characters between the undescribed taxon and its congeners. Therefore, adopt integrative taxonomy approaches with evidence from molecular and morphology to take the decision to describe the unidentified taxon as new species described herein.

**Table 3.** The results of the one-way ANOVA with P-values for morphometric comparisons between males of *Odorranaleishanensis* sp. nov., *O.hejiangensis*, *O.huanggangensis*, *O.ichangensis*, *O.kweichowensis*, *O.schmackeri*, and *O.wuchuanensis*.

	OL vs OHG	OL vs OHJ	OL vs OI	OL vs OK	OL vs OS	OL vs OW
SVL	0.841	0.000**	0.006**	0.193	0.193	0.000**
HDL	0.001**	0.020*	0.000**	0.003**	0.003**	0.001**
HDW	0.643	0.967	0.599	0.469	0.469	0.000**
SL	0.192	0.577	0.044*	0.495	0.495	0.011*
NED	0.364	0.313	0.185	0.394	0.394	0.094
NSD	0.002**	0.067	0.011*	0.145	0.145	0.002**
IND	0.054	0.000**	0.000**	0.000**	0.000**	0.157
ED	0.005**	0.015*	0.067	0.015*	0.015*	0.128
IOD	0.164	0.002**	0.586	0.016*	0.016*	0.409
UEW	0.006**	0.018*	0.223	0.009**	0.009**	0.934
TYD	0.000**	0.000**	0.000**	0.000**	0.000**	0.000**
LAL	0.016*	0.007**	0.000**	0.001**	0.001**	0.000**
LW	0.163	0.000**	0.007**	0.001**	0.001**	0.009**
ML	0.801	0.237	0.000**	0.029*	0.029*	0.852
HLL	0.197	0.022*	0.001**	0.230	0.230	0.660
THL	0.406	0.021*	0.020*	0.745	0.745	0.450
TL	0.524	0.224	0.283	0.173	0.173	0.049*
TW	0.272	0.000**	0.005**	0.036*	0.036*	0.414
FL	0.003**	0.007**	0.036*	0.025*	0.025*	0.001**
TFL	0.505	0.812	0.343	0.583	0.583	0.622

Notes: OL, *Odorranaleishanensis* sp. nov.; OHG, *O.huanggangensis*; OHJ, *O.hejiangensis*; OI, *O.ichangensis*; OK, *O.kweichowensis*; OS, *O.schmackeri*; OW, *O.wuchuanensis*. Significance level: * p < 0.05; ** p < 0.01. Abbreviations for the morphometric characters refer to Materials and methods section.

### ﻿Taxonomic accounts

#### ﻿ Odorrana leishanensis sp. nov.

Taxon classificationAnimaliaAnuraRanidae

A183195B-624D-56E4-811C-69FC6532EFC7

https://zoobank.org/D51EC9FE-C269-4189-9815-AB65D3FBE0B6

Figs 4 , 5 , 6

##### Material examined.

***Holotype*.**MT LS20230729013, adult male, collected by Jing Liu on 29 July 2023 in the Leigongshan Nature Reserve (26.3833°N, 108.1967°E; elevation 1830 m a.s.l.), Leishan County, Guizhou Province, China. ***Paratype*.** Two males MT LS20230711020 and MT LS20230717001, collected by Jing Liu on 11 and 17 July 2023; one male MT LS20230805001 collected by Chaobo Feng on 5 August 2023; two males MT LS20230806010, MT LS20230806018 and one female MT LS20230811024 collected by Shize Li on 6 and 8 August 2023 from the same place as holotype.

##### Diagnosis.

*Odorranaleishanensis* sp. nov. can be distinguished from its congeners by the following characters: (1) body size moderate (SVL♂ (*n* = 6) = 39.1–49.4 mm, SVL♀ (*n* = 1) = 49.7 mm in female); (2) head width larger than head length; (3) tympanum distinctly visible; (4) small rounded granules scattered all over dorsal body and limbs; (5) dorsolateral folds absent; (6) heels overlapping when thighs are positioned at right angles to the body; tibiotarsal articulation reaching the level between eye to nostril when leg stretched forward; (7) vocal sacs in male absent, and nuptial pads in male present on base of finger I.

##### Description of holotype

**Figure 4.**
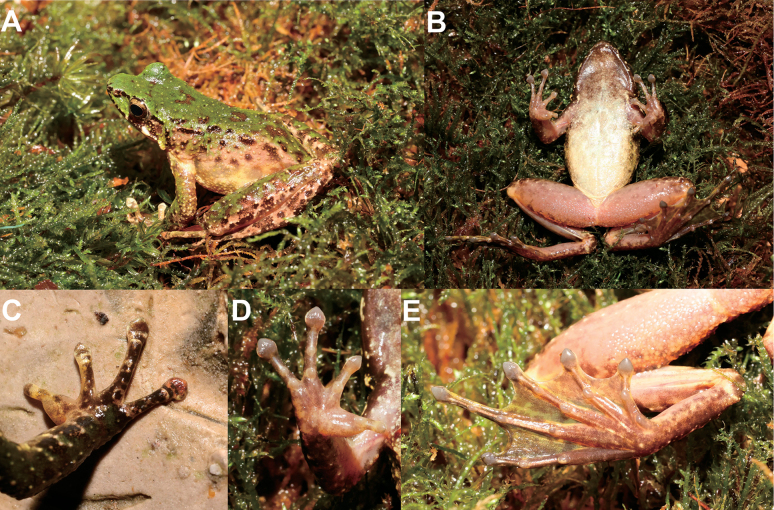
Photographs of the holotype MTLS20230729013 of *Odorranaleishanensis* sp. nov. in life **A** dorsal view **B** ventral view **C** dorsal view of hand **D** ventral view of hand **E** ventral view of foot.

**Figure 5.**
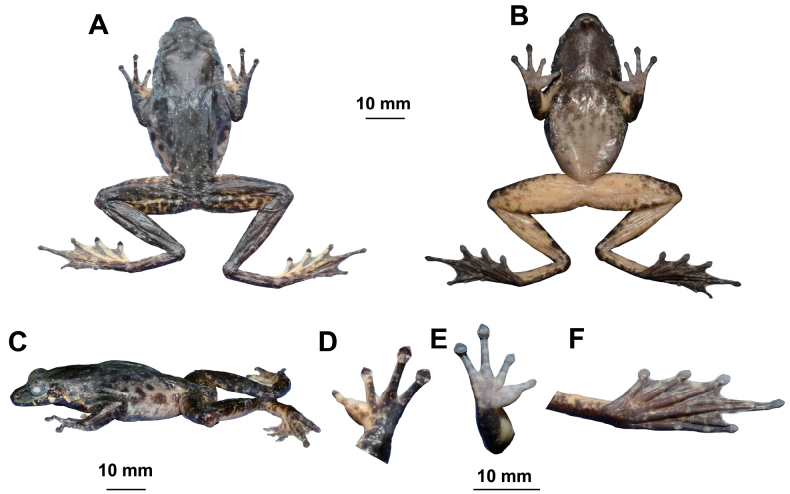
The holotype specimen MTLS20230729013 of *Odorranaleishanensis* sp. nov. (preserved) in **A** dorsal view **B** ventral view **C** lateral view **D** dorsal view of hand **E** ventral view of hand **F** ventral view of foot.

**(Figs 4, 5).** Adult male, body size moderate (SVL 49.4 mm); head width larger than head length (HDW/HDL = 1.14); snout short, rounded in dorsal view, projecting beyond lower jaw; eye large and convex, ED 0.73 SL; nostril rounded, closer to tip of snout than to eye; internasal distance larger than interorbital distance; tympanum distinct, approximately 0.68 ED; vomerine teeth on well-developed ridges; tongue deeply notched posteriorly; pupil horizontally oval; vocal sac absent.

Forelimbs slender (LW/SVL = 0.09); lower arm and hand not reach one-second of body length (LAL/SVL = 0.42); fingers slender, relative finger lengths II < I < IV < III; finger tips on I–IV dilated to wide cordiform disks with circum-marginal grooves, without webbing and lateral fringes; subarticular tubercle prominent; supernumerary tubercle indistinct; inner metacarpal tubercle oval, elongate; outer metacarpal tubercles absent; light yellow glandular nuptial pad on finger I.

Hindlimbs long; tibio-tarsal articulation reaching between eye to nostril when hindlimb adpressed along the side of the body; heels overlapped; tibia longer than thigh length; toes slender, relative lengths I < II < III < V < IV; toes entirely webbed; tips of toes expanded into disc with circummarginal grooves; outer metatarsal tubercle absent; inner metatarsal tubercle present.

Dorsal rough, there are small, rounded granules scattered all over dorsal body and limbs, ventral surfaces of the head, body, and limbs smooth; weak supratympanic fold from the posterior edge of the eye to the posterior edge of the tympanum; dorsolateral folds absent.

##### Coloration of holotype in life

**(Fig. 4).** Dorsum grass-green with a small amount of brown spots; flanks pale yellow with several black spots; dorsal surfaces of anterior forelimbs pale yellow, anterior forelimbs olive-brown, with black bands and irregular grass-green spots; dorsal surfaces of hindlimbs grass-green with black bands; upper jaw with a ring of brown spots; lower jaw yellow with black spots; grass-green and black spotted mosaic on the loreal region; tympanum brown-black; ventral surface of throat and chest brown, belly pale yellow.

##### Coloration of holotype in preservation

**(Fig. 5).** After three months in 75% ethanol, the dorsal surface of the body faded to dark olive; the dorsal surface of the head changed to darker; the transverse bands on limbs and digits were not distinct; ventral surface of throat brown, gradually dark brown on chest, the belly was pale yellow; palm color faded to white.

##### Variation.

**Figure 6.**
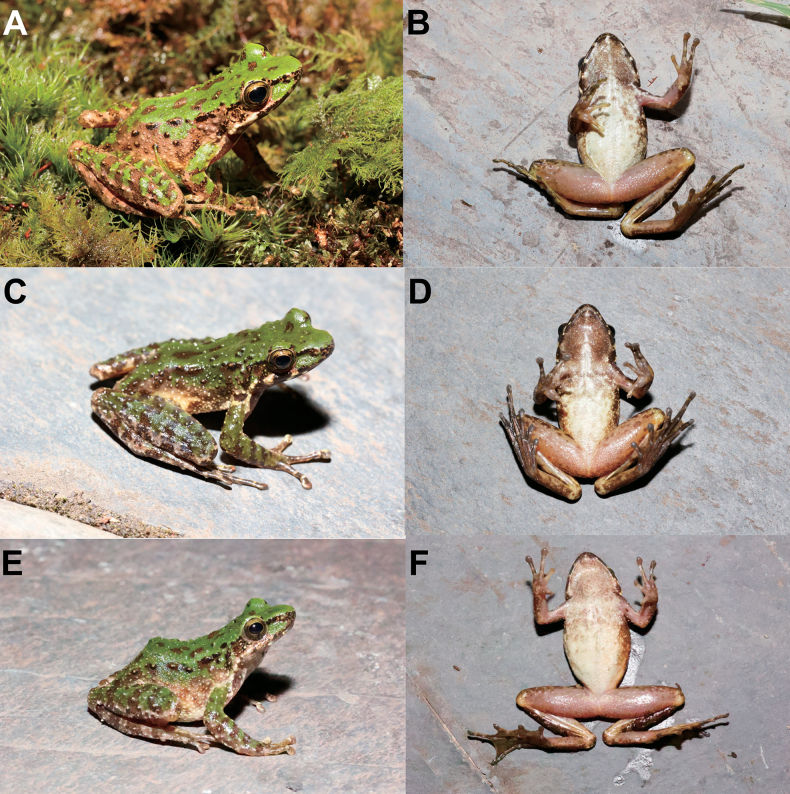
Color variation in *Odorranaleishanensis* sp. nov. **A** dorsolateral view of the male specimen MTLS20230805001 **B** ventral view of the male specimen MTLS20230805001 **C** dorsolateral view of the male specimen LS20230806010 **D** ventral view of the male specimen MTLS20230806010 **E** dorsolateral view of the female specimen LS20230811024 **F** ventral view of the female specimen MTLS20230811024.

Morphological measurements of all specimens are presented in Table 4 and Suppl. material 1. All specimens were very similar in morphology and color pattern, but in MT LS20230805001 the skin from the corner of the eye to the base of the thigh was noticeably pale brown with green patches mixed in and the flank of the ventral surface was white with dark brown spots (Fig. 6A, B); in MT LS20230806010 the dorsum was green and the ventral surface of the throat and chest darker (Fig. 6C, D); in MT LS20230811024 the granulation on the dorsolateral surface was covered with black spots and the ventral surface of the throat and chest were white with darker spots (Fig. 6E, F).

**Table 4.** Measurements of the adult specimens of *Odorranaleishanensis* sp. nov. Units are given in mm. See abbreviations for the morphological characters in Materials and methods section.

Character	Holotype	Males (*n* = 6)		Female (*n* = 1)
		Range	Mean ± SD	
SVL	49.43	39.1–49.4	42.3 ± 4.0	49.7
HDL	15.13	11.6–17.8	13.7 ± 2.2	17.2
HDW	17.23	13.3–17.2	14.6 ± 1.4	17.7
SL	6.73	5.6–6.7	6.2 ± 0.5	7.7
NED	3.57	2.4–3.6	3.2 ± 0.4	4.1
NSD	2.92	1.6–2.9	2.4 ± 0.5	3.4
IND	6.02	4.8–6.0	5.3 ± 0.5	6.3
ED	4.94	4.1–4.9	4.5 ± 0.3	5.8
IOD	3.76	3.4–4.4	4.0 ± 0.3	5.1
UEW	4.04	2.9–4.0	3.4 ± 0.4	4.8
TYD	3.36	2.3–3.4	2.5 ± 0.4	2.5
LAL	20.52	17.4–20.5	18.7 ± 1.2	24.6
LW	4.38	2.9–4.4	3.3 ± 0.6	4.7
ML	12.19	10.5–12.2	11.3 ± 0.6	14.9
HLL	82.28	67.8–82.3	72.3 ± 5.2	87.3
THL	24.57	19.9–24.6	21.2 ± 1.7	28.3
TL	27.65	22.8–27.7	24.5 ± 1.75	29.7
TW	5.77	3.5–5.8	4.4 ± 0.7	6.9
TFL	38.43	31.3–38.4	33.8 ± 2.6	39.6
FL	26.66	22.7–26.7	23.9 ± 1.5	28.2

##### Secondary sexual characters.

Adult females slightly larger than adult males; adult males lack vocal sacs. During breeding season, pale yellow glandular nuptial pads in males present on finger I (Figs 4C, 5D).

##### Comparisons.

The molecular phylogenetic analyses placed the new species in an independent clade and sharing a sister relationship with the clade composed of *O.schmackeri*, *O.kweichowensis*, *O.fengkaiensis*, *O.hainanensis*, *O.bacboensis*, *O.ichangensis*, *O.hejiangensis*, *O.tianmuii*, and *O.huanggangensis*. *Odorranaleishanensis* sp. nov. differs from the aforementioned species by having a similar body size in males and females, SVL♂ = 39.1–49.4 mm, ♀ = 49.7 mm) (vs female size larger than males); vocal sac in males absent (vs present).

*Odorranaleishanensis* sp. nov. differs from *O.amamiensis*, *O.andersonii*, *O.aureola*, *O.bacboensis*, *O.cangyuanensis*, *O.chapaensis*, *O.chloronota*, *O.damingshanensis*, *O.geminata*, *O.grahami*, *O.ishikawae*, *O.indeprensa*, *O.jingdongensis*, *O.junlianensis*, *O.kuangwuensis*, *O.leporipes*, *O.lungshengensis*, *O.mutschmanni*, *O.nanjiangensis*, *O.narina*, *O.splendida*, *O.supranarina*, *O.tiannanensis*, *O.versabilis*, and *O.wuchuanensis* in having a medium body size (maximum SVL < 50.0 mm in males vs minimum SVL > 50.0 mm in all other species).

*Odorranaleishanensis* sp. nov. differs from *O.absita*, *O.amamiensis*, *O.andersonii*, *O.anlungensis*, *O.aureola*, *O.bacboensis*, *O.banaorum*, *O.bolavensis*, *O.chapaensis*, *O.chloronota*, *O.dulongensis*, *O.fengkaiensis*, *O.geminata*, *O.grahami*, *O.graminea*, *O.hainanensis*, *O.heatwolei*, *O.hejiangensis*, *O.hosii*, *O.huanggangensis*, *O.ichangensis*, *O.indeprensa*, *O.jingdongensis*, *O.junlianensis*, *O.khalam*, *O.kuangwuensis*, *O.kweichowensis*, *O.liboensis*, *O.livida*, *O.lungshengensis*, *O.macrotympana*, *O.margaretae*, *O.monjerai*, *O.morafkai*, *O.mutschmanni*, *O.nanjiangensis*, *O.narina*, *O.orba*, *O.sangzhiensis*, *O.schmackeri*, *O.sinica*, *O.splendida*, *O.supranarina*, *O.swinhoana*, *O.tiannanensis*, *O.tormota*, *O.versabilis*, *O.wuchuanensis*, *O.yentuensis*, *O.yizhangensis*, and *O.yunnanensis* by having medium female body size (SVL < 50.0 mm vs minimum SVL > 50.0 mm in the other species).

*Odorranaleishanensis* sp. nov. differs from *O.absita*, *O.amamiensis*, *O.banaorum*, *O.bolavensis*, *O.chloronota*, *O.confusa*, *O.damingshanensis*, *O.exiliversabilis*, *O.gigatympana*, *O.graminea*, *O.heatwolei*, *O.hosii*, *O.khalam*, *O.leporipes*, *O.livida*, *O.macrotympana*, *O.margaretae*, *O.monjerai*, *O.narina*, *O.nasica*, *O.nasuta*, *O.orba*, *O.supranarina*, *O.tormota*, *O.utsunomiyaorum*, *O.versabilis* and *O.yentuensis* by lacking dorsolateral folds (vs present in the other species).

*Odorranaleishanensis* sp. nov. differs from *O.bacboensis*, *O.jingdongensis*, *O.lungshengensis*, *O.margaretae*, *O.mutschmanni*, *O.nanjiangensis*, *O.narina*, *O.orba*, *O.sinica*, *O.swinhoana*, *O.tormota*, and *O.yizhangensis* by the tibiotarsal articulation reaching to between the eye and the nostril when the leg is stretched forward (vs reaching the tip of the snout), from *O.nasica* and *O.nasuta* (vs reaching the tip of the snout or a little beyond), from *O.hainanensis* (vs reaching the tip of the snout or the anterior corner of eye), from *O.junlianensis* (vs reaching the tip of the snout or between the nostril and the snout), from *O.cangyuanensis*, *O.exiliversabilis*, *O.fengkaiensis*, *O.gigatympana*, *O.grahami*, *O.graminea*, *O.tiannanensis*, *O.versabilis*, and *O.yentuensis* (vs reaching to or beyond the tip of the snout), from *O.amamiensis* (vs reaching far beyond the tip of the snout), from *O.amamiensis*, *O.anlungensis*, *O.huanggangensis*, *O.kuangwuensis*, *O.macrotympana*, *O.wuchuanensis*, and *O.ichangensis* (vs reaching the nostril or beyond the tip of the snout), from *O.lipuensis*, *O.splendida*, and *O.supranarina* (vs reaching the anterior corner of the eye), and from *O.utsunomiyaorum* (vs reaching between the anterior corner of the eye and the nostril).

*Odorranaleishanensis* sp. nov. differs from *O.absita*, *O.amamiensis*, *O.andersonii*, *O.anlungensis*, *O.aureola*, *O.bacboensis*, *O.banaorum*, *O.bolavensis*, *O.cangyuanensis*, *O.chapaensis*, *O.chloronota*, *O.dulongensis*, *O.exiliversabilis*, *O.fengkaiensis*, *O.geminata*, *O.gigatympana*, *O.grahami*, *O.graminea*, *O.hainanensis*, *O.heatwolei*, *O.hejiangensis*, *O.huanggangensis*, *O.ichangensis*, *O.indeprensa*, *O.ishikawae*, *O.jingdongensis*, *O.junlianensis*, *O.khalam*, *O.kweichowensis*, *O.lungshengensis*, *O.macrotympana*, *O.morafkai*, *O.nanjiangensis*, *O.nasica*, *O.nasuta*, *O.orba*, *O.sinica*, *O.swinhoana*, *O.tianmuii*, *O.tiannanensis*, *O.tormota*, *O.utsunomiyaorum*, *O.versabilis*, *O.yentuensis* and *O.yizhangensis* by vocal sacs absent in male (vs present in the other species).

The congeners *O.graminea*, *O.huanggangensis*, and *O.lungshengensis* have sympatric distribution with *Odorranaleishanensis* sp. nov. (Fei et al. 2012; Amphibia China 2024). The new species can be distinguished from these species by a series of morphological characters as follows. This new species differs from *O.graminea* by the presence of vocal sacs in male and dorsolateral folds absent (vs vocal sacs in male and dorsolateral folds present in the latter) and small, rounded but rough dorsal granules scattered all over dorsal body and limbs (vs dorsum smooth in the latter). It differs from *O.huanggangensis* and *O.lungshengensis* by vocal sacs in male absent (vs vocal sacs present in male in the latter) and small, rounded but rough dorsal granules scattered all over dorsal body and limbs (vs dorsum smooth the other species).

##### Distribution and habitats.

**Figure 7.**
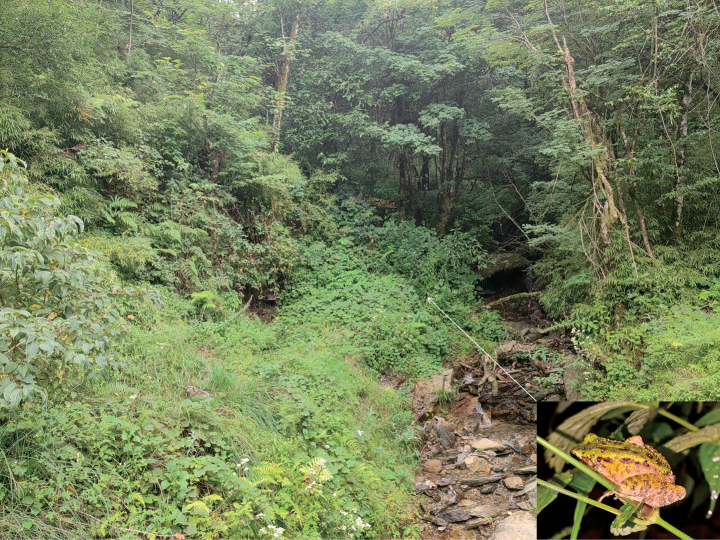
Habitats of *Odorranaleishanensis* sp. nov. at the type locality, Leishan County, Guizhou Province, China (inset: the holotype on bush stems beside the stream).

At present, *Odorranaleishanensis* sp. nov. is only known from Leigongshan National Nature Reserve, Leishan County, Guizhou Province, China. The population inhabits mountain forest at elevations between 1600–1800 m and is often found on bamboo and encountered in forest nearby streams (Fig. 7). *Boulenophrysleishanensis* Li, Xu, Liu, Jiang, Wei & Wang, 2018, *B.spinata* Liu & Hu, 1973, *O.lungshengensis* Liu & Hu, 1962, *Leptobrachellawulingensis* Qian, Xia, Cao, Xiao & Yang, 2020, *Paramesotritoncaudopunctatus* Liu & Hu, 1973 and *Leptobrachiumleishanensis* Liu & Hu, 1973, were also found in the type locality of the new species.

##### Etymology.

The specific epithet *leishanensis* refers to the distribution of this species, Leishan County, Guizhou Province, China. We propose the common English name “Leishan Odorous Frog” and the Chinese name as “Lei Shan Chou Wa (雷山臭蛙)” for this species.

## ﻿Discussion

In recent years, new species of *Odorrana* have been discovered almost every year (Frost 2024). Within the genus, *O.schmackeri* has been considered as the most widespread species in China, covering Henan, Sichuan, Chongqing, Guizhou, Hubei, Anhui, Jiangsu, Zhejiang, Hunan, Fujian, Guangdong, and Guangxi provinces (Fei et al. 2012). In recent years *O.schmackeri* was indicated as a complex of species, probably containing some cryptic species (Chen et al. 2013; Li et al. 2015; Zhu 2016), and have been described one after another (Wang et al. 2015; Li et al. 2018a; Shen et al. 2020; Zhang et al. 2021). Molecular phylogenetic analyses indicated that *Odorranaleishanensis* sp. nov. was revealed as the sister to the clade corresponding to the *O.schmackeri* complex, and is morphologically distinct from the latter (vocal sacs absent, and smaller body size in female). This may indicate that the new species has probably experienced an independent evolutionary history.

Leigong Mountain in Guizhou Province, China is the main summit of the Miaoling mountain range. Since the 1980s, many scholars have investigated the amphibians in this area and several species were described, i.e., *Paramesotritoncaudopunctatus* (Liu & Hu, 1973), *Boulenophrysspinata*, *Leptobrachiumleishanense* (Liu & Hu, 1973), *B.leishanensis*, and *Nidiranaleishanensis* Li, Wei, Xu, Cui, Fei, Jiang, Liu & Wang, 2019. Among them, *B.leishanensis* and *N.leishanensis* had previously been misidentified as *B.minor* (Stejneger, 1926) and *N.adenopleura* (Boulenger, 1909)(Hu et al. 1973; Li et al. 2018b; Li et al. 2019). From 2014 to July 2023 we conducted several surveys in this region but the new species has only just been discovered, with only seven adult specimens found in a small area at elevations of 1600–1800 m. Therefore, we infer that the population of the new species is small, and we recommend classifying the new species as vulnerable (VU) according to the evaluation criteria of the IUCN Red List of threatened Species (IUCN 2012). Future research should focus on determining the distribution and elevational range of the species.

## Supplementary Material

XML Treatment for Odorrana leishanensis

## ﻿Additional information

### Author ORCIDs

Bin Wang https://orcid.org/0000-0001-6036-5579

### Data availability

All of the data that support the findings of this study are available in the main text or Supplementary Information.

## Supplementary materials

Supplementary material 1 Measurements of the adult specimens of *Odorranaleishanensis* sp. nov., *O.hejiangensis*, *O.huanggangensis*, *O.ichangensis*, *O.kweichowensis*, *O.schmackeri*, and *O.wuchuanensis* Data type xlsx Explanation note Units in mm. See abbreviations for the morphological characters in Materials and methods section. https://binary.pensoft.net/file/986533This dataset is made available under the Open Database License (http://opendatacommons.org/licenses/odbl/1.0/). The Open Database License (ODbL) is a license agreement intended to allow users to freely share, modify, and use this Dataset while maintaining this same freedom for others, provided that the original source and author(s) are credited.Shi-Ze Li, Ji-Jun Chen, Hai-Jun Su, Jing Liu, Xiu-Jun Tang, Bin Wang

Supplementary material 2 Uncorrected *p*-distances between the *Odorrana* species based on the *12S rRNA* gene sequences Data type xlsx https://binary.pensoft.net/file/986534This dataset is made available under the Open Database License (http://opendatacommons.org/licenses/odbl/1.0/). The Open Database License (ODbL) is a license agreement intended to allow users to freely share, modify, and use this Dataset while maintaining this same freedom for others, provided that the original source and author(s) are credited.Shi-Ze Li, Ji-Jun Chen, Hai-Jun Su, Jing Liu, Xiu-Jun Tang, Bin Wang

Supplementary material 3 Uncorrected *p*-distances between the *Odorrana* species based on the 1*6S rRNA* gene sequences Data type xlsx https://binary.pensoft.net/file/986535This dataset is made available under the Open Database License (http://opendatacommons.org/licenses/odbl/1.0/). The Open Database License (ODbL) is a license agreement intended to allow users to freely share, modify, and use this Dataset while maintaining this same freedom for others, provided that the original source and author(s) are credited.Shi-Ze Li, Ji-Jun Chen, Hai-Jun Su, Jing Liu, Xiu-Jun Tang, Bin Wang

Supplementary material 4 Uncorrected *p*-distances between the *Odorrana* species based on the *ND2* gene sequences Data type xls https://binary.pensoft.net/file/986536This dataset is made available under the Open Database License (http://opendatacommons.org/licenses/odbl/1.0/). The Open Database License (ODbL) is a license agreement intended to allow users to freely share, modify, and use this Dataset while maintaining this same freedom for others, provided that the original source and author(s) are credited.Shi-Ze Li, Ji-Jun Chen, Hai-Jun Su, Jing Liu, Xiu-Jun Tang, Bin Wang

Supplementary material 5 Maximum likelihood (ML) tree of the genus *Odorrana* reconstructed based on the *12S rRNA* gene sequences Data type jpg Explanation note ML bootstrap supports (BS)/Bayesian posterior probability (BPP) are denoted beside each node, and “-” denotes BS < 50% or BPP < 0.60. Samples 1–86 refer to those listed in Table 1. https://binary.pensoft.net/file/986537This dataset is made available under the Open Database License (http://opendatacommons.org/licenses/odbl/1.0/). The Open Database License (ODbL) is a license agreement intended to allow users to freely share, modify, and use this Dataset while maintaining this same freedom for others, provided that the original source and author(s) are credited.Shi-Ze Li, Ji-Jun Chen, Hai-Jun Su, Jing Liu, Xiu-Jun Tang, Bin Wang

Supplementary material 6 Maximum likelihood (ML) tree of the genus *Odorrana* reconstructed based on the *16S rRNA* gene sequences Data type jpg Explanation note ML bootstrap supports (BS)/Bayesian posterior probability (BPP) are denoted beside each node, and “-” denotes BS < 50% or BPP < 0.60. Samples 1–86 refer to those listed in Table 1. https://binary.pensoft.net/file/986538This dataset is made available under the Open Database License (http://opendatacommons.org/licenses/odbl/1.0/). The Open Database License (ODbL) is a license agreement intended to allow users to freely share, modify, and use this Dataset while maintaining this same freedom for others, provided that the original source and author(s) are credited.Shi-Ze Li, Ji-Jun Chen, Hai-Jun Su, Jing Liu, Xiu-Jun Tang, Bin Wang

Supplementary material 7 Maximum likelihood (ML) tree of the genus *Odorrana* reconstructed based on the *ND2* gene sequences Data type jpg Explanation note ML bootstrap supports (BS)/Bayesian posterior probability (BPP) were denoted beside each node, and “-” denotes BS < 50% or BPP < 0.60. Samples 1–86 refer to those listed in Table 1. https://binary.pensoft.net/file/986539This dataset is made available under the Open Database License (http://opendatacommons.org/licenses/odbl/1.0/). The Open Database License (ODbL) is a license agreement intended to allow users to freely share, modify, and use this Dataset while maintaining this same freedom for others, provided that the original source and author(s) are credited.Shi-Ze Li, Ji-Jun Chen, Hai-Jun Su, Jing Liu, Xiu-Jun Tang, Bin Wang

## References

[B1] Ahl E (1927 [1925]) Über vernachlässigte Merkmale bei Fröschen. Sitzungsberichte der Gesellschaft Naturforschender Freunde zu Berlin, 40–47.

[B2] Amphibia China (2024) The database of Chinese amphibians. Electronic Database. Kunming Institute of Zoology (CAS), Kunming, Yunnan. http://www.amphibiachina.org/ [Accessed 24 January 2024]

[B3] Bain RH Stuart BL (2006 [2005]) A new species of cascade frog (Amphibia: Ranidae) from Thailand, with new data on *Ranabanaorum* and *Ranamorafkai*. The Natural History Bulletin of the Siam Society 53(1): 3–16. 10.1643/0045-8511(2006)006[0043:TNISOC]2.0.CO;2

[B4] Bain RH Lathrop A Murphy RW Orlov NL Cuc HT (2003) Cryptic species of a cascade frog from Southeast Asia: Taxonomic revisions and descriptions of six new species. American Museum Novitates 3417: 1–60. 10.1206/0003-0082(2003)417<0001:CSOACF>2.0.CO;2

[B5] Bain RH Stuart BL Nguyen TQ Che J Rao DQ (2009) A new *Odorrana* (Amphibia: Ranidae) from Vietnam and China. Copeia 2(2): 348–362. 10.1643/CH-07-195

[B6] Blyth E (1856) Report for October Meeting, 1855. Journal of the Asiatic Society of Bengal 24: 711–723.

[B7] Boettger O (1892) Katalog der Batrachier-Sammlung im Museum der Senckenbergischen Naturforschenden Gesellshaft in Frankfurt am Main. Gebrüder Knauer, Frankfurt am Main, 11 pp.

[B8] Boulenger GA (1882) Calalogue of the Batrachia Salientias. Ecaudata in the collection of the British Museum, London, 55 pp.

[B9] Boulenger GA (1891) On new or little-known Indian and Malayan reptiles and batrachians. Annals & Magazine of Natural History 8(46): 288–292. 10.1080/00222939109460437

[B10] Boulenger GA (1900) On the reptiles, batrachians, and fishes collected by the late Mr. John Whitehead in the interior of Hainan. Proceedings of the Zoological Society of London 1899: 956–962.

[B11] Boulenger GA (1903) Descriptions of new batrachians in the British Museum. Annals & Magazine of Natural History 12(71): 552–557. 10.1080/00222930308678892

[B12] Boulenger GA (1917) Descriptions of new frogs of the genus *Rana*. Annals & Magazine of Natural History 20(120): 413–418. 10.1080/00222931709487029

[B13] Bourret R (1937) Notes herpétologiques sur l’Indochine française. XIV. Les batraciens de la collection du Laboratoire des Sciences naturelles de l’Université. Descriptions de quinze espèces ou variétés nouvelles. Annexe au Bulletin Général de l’Instruction Publique 4: 5–56.

[B14] Che J Pang JF Zhao H Wu GF Zhao EM Zhang YP (2007) Phylogeny of Raninae (Anura: Ranidae) inferred from mitochondrial and nuclear sequences. Molecular Phylogenetics and Evolution 43(1): 1–13. 10.1016/j.ympev.2006.11.032 17300963

[B15] Che J Jiang K Yan F Zhang YP (2020) Amphibians and Reptiles in Tibet – Diversity and Evolution. Science Press, Beijing, 238–243.

[B16] Chen LQ Murphy RW Lathrop A Ngo A Orlov NL Ho CT Somorjai I (2005) Taxonomic chaos in Asian ranid frogs: An initial phylogenetic resolution. The Herpetological Journal 15: 231–243.

[B17] Chen XH Zhou KY Zheng GM (2010a) A new species of the genus *Odorrana* from China (Anura, Ranidae). Dong Wu Fen Lei Xue Bao 35(1): 206–211.

[B18] Chen XH Zhou KY Zheng GM (2010b) A new species of odorous frog from China (Anura: Ranidae). Journal of Beijing Normal University 46(5): 606–609.

[B19] Chen XH Chen Z Jiang JP Qiao L Lu YQ Zhou KY Zheng GM Zhai XF Liu JX (2013) Molecular phylogeny and diversification of the genus *Odorrana* (Amphibia, Anura, Ranidae) inferred from two mitochondrial genes. Molecular Phylogenetics and Evolution 69(3): 1196–1202. 10.1016/j.ympev.2013.07.023 23911727

[B20] Chen WC Mo YM Lin L Qin K (2024) A new species of *Odorrana* Fei, Ye & Huang, 1990 (Amphibia, Anura, Ranidae) from central Guangxi, China with a discussion of the taxonomy of Odorrana (Bamburana). ZooKeys 1190: 131–152. 10.3897/zookeys.1190.109886 38313454 PMC10835719

[B21] Deng QX Yu ZW (1992) A new species of the genus *Rana* form China. Journal of Sichuan Teacher College 13(4): 323–326.

[B22] Fei L Ye CY Huang YZ (1990) Key to Chinese Amphibians. Publishing House for Scientific and Technological Literature, Chongqing, 364 pp.

[B23] Fei L Ye CY Li C (2001a) Descriptions of two new species of the genus *Odorrana* in China (Anura: Ranidae). Dong Wu Fen Lei Xue Bao 26(1): 108–114.

[B24] Fei L Ye CY Li C (2001b) Taxonomic studies of *Odorranaversabilis* in China II. Descriptions of two new species (Amphibia: Ranidae). Dong Wu Fen Lei Xue Bao 26(4): 601–607.

[B25] Fei L Ye CY Jiang JP Xie F Huang YZ (2005) An Illustrated Key to Chinese Amphibians. Sichuan Publishing House of Science and Technology, Chongqing, 123 pp.

[B26] Fei L Ye CY Jiang JP (2007a) A new Ranidae frog species China Odorrana (Odorrana) yizhangensis (Ranidae: Anura). Dong Wu Fen Lei Xue Bao 32(4): 989–992.

[B27] Fei L Ye CY Xie F Jiang JP (2007b) A new Ranidae frog species from Sichuan, China Odorrana (Odorrana) nanjiangensis (Ranidae: Anura). Zoological Research 28(5): 551–555.

[B28] Fei L Hu SQ Ye CY Huang YZ (2009) Fauna Sinica. Amphibia (Vol. 2) Anura. Science Press, Beijing, 957 pp.

[B29] Fei L Ye CY Jiang JP (2012) Colored Atlas of Chinese Amphibians and Their Distributions. Sichuan Publishing House of Science & Technology, Chengdu, 619 pp.

[B30] Frost DR (2024) Amphibian Species of the World: an Online Reference. Version 6.1. Electronic Database. American Museum of Natural History, New York. http://research.amnh.org/herpetology/amphibia/index.html [Accessed 24 January 2024]

[B31] Frost DR Grant T Faivovich J Bain RH Haas A Haddad CFB de Sá RO Channing A Wilkinson M Donnellan SC Raxworthy CJ Campbell JA Blotto BL Moler PE Drewes RC Nussbaum RA Lynch JD Green DM Wheeler WC (2006) The amphibian tree of life. Bulletin of the American Museum of Natural History 297: 1–370. 10.1206/0003-0090(2006)297[0001:TATOL]2.0.CO;2

[B32] Guindon S Dufayard JF Lefort V Anisimova M Hordijk W Gascuel O (2010) New algorithms and methods to estimate maximum-likelihood phylogenies: assessing the performance of PhyML 3.0. Systematic Biology 59(3): 07–321. 10.1093/sysbio/syq010 20525638

[B33] Günther A (1876) Tird report on collections of Indian reptiles obtained by the British Museum. Proceedings of the Zoological Society of London 1875: 567–577.

[B34] Hall TA (1999) BIOEDIT: A user-friendly biological sequence alignment editor and analysis program for Windows 95/98/NT. Nucleic Acids Symposium Series 41(41): 95–98.

[B35] Hu SX Zhao EM Liu CZ (1966) A herpetological survey of the Tsinling and Ta-pa shan Regions. Acta Zoological Sinica 18(1): 57–89.

[B36] Hu SX Zhao EM Liu CZ (1973) A survey of amphibians and reptiles in Kweichow Province, including a herpetofauna analysis. Acta Zoological Sinica 19(2): 149–171.

[B37] IUCN (2012) IUCN Red List Categories and Criteria: Version 3.1, (2^nd^ edn.). Cambridge, Gland, 16 pp.

[B38] Kocher TD Thomas WK Meyer A Edwards SV Paabo S Villablanca FX Wilson AC (1989) Dynamics of mitochondrial DNA evolution in mammals: Amplification and sequencing with conserved Primers. Proceedings of the National Academy of Sciences of the United States of America 86(16): 6169–6200. 10.1073/pnas.86.16.6196 PMC297804 2762322

[B39] Kurabayashi A Natsuhiko Y Naoki S Yoko H Shohei O Tamotsu F Masayuki S (2010) Complete mitochondrial DNA sequence of the endangered frog *Odorranaishikawae* (family Ranidae) and unexpected diversity of mt gene arrangements in ranids. Molecular Phylogenetics and Evolution 56(2): 543–553. 10.1016/j.ympev.2010.01.022 20102742

[B40] Kuramoto M Satou N Oumi S Kurabayashi A Sumida M (2011) Inter-and intra-Island divergence in *Odorranaishikawae* (Anura, Ranidae) of the Ryukyu Archipelago of Japan, with description of a new species. Zootaxa 2767(1): 25–40. 10.11646/zootaxa.2767.1.3

[B41] Lanfear R Calcott B Ho SYW Guindon S (2012) PartitionFinder: Combined selection of partitioning schemes and substitution models for phylogenetic analyses. Molecular Biology and Evolution 29(6): 1695–1701. 10.1093/molbev/mss020 22319168

[B42] Li YM Wu XY Zhang HB Yan P Xue H Wu XB (2015) Vicariance and its impact on the molecular ecology of a Chinese Ranid frog species-complex (*Odorranaschmackeri*, Ranidae). PLOS ONE 10(9): e0138757. 10.1371/journal.pone.0138757 PMC4578928 26394403

[B43] Li YM Wu XY Zhang HB Yan P Xue H Wu XB (2016) The complete mitochondrial genome of *Amolopsricketti* (Amphidia, Anura, Ranidae). Mitochondrial DNA. Part A, DNA Mapping, Sequencing, and Analysis 27(1): 242–243. 10.3109/19401736.2014.883606 24521496

[B44] Li SZ Xu N Lv JC Jiang JP Wei G Wang B (2018a) A new species of the odorous frog genus *Odorrana* (Amphibia, Anura, Ranidae) from southwestern China. PeerJ 6: e5695. 10.7717/peerj.5695 PMC6174872 30310744

[B45] Li SZ Xu N Liu J Jiang JP Wei G Wang B (2018b) A new species of the Asian toad genus *Megophrys* sensu lato (Amphibia: Anura: Megophryidae) from Guizhou Province, China. Asian Herpetological Research 9(4): 224–239. 10.16373/j.cnki.ahr.180072

[B46] Li SZ Wei G Xu N Cui JG Fei L Jiang JP Liu J Wang B (2019) A new species of the Asian music frog genus *Nidirana* (Amphibia, Anura, Ranidae) from Southwestern China. PeerJ 7: e7157. 10.7717/peerj.7157 PMC6174872 30310744

[B47] Lin SS Li YH Su HL Yi H Pan Z Sun YJ Zeng ZC Wang J (2022) Discovery of a new limestone karst-restricted odorous frog from northern Guangdong, China (Anura, Ranidae, *Odorrana*). ZooKeys 1120: 47–66. 10.3897/zookeys.1120.87067 36760328 PMC9848673

[B48] Liu CZ (1950) Amphibians of western China. Fieldiana. Zoology Memoirs 2: 1–400. 10.5962/bhl.part.4737

[B49] Liu CZ Hu SQ (1962) A survey of amphibians and reptiles in Guangxi Province. Acta Zoological Sinica 14: 73–104.

[B50] Liu XL He YH Wang YF Beukema W Hou SB Li YC Che J Yuan ZY (2021) A new frog species of the genus *Odorrana* (Anura: Ranidae) from Yunnan, China. Zootaxa 4908(2): 263–275. 10.11646/zootaxa.4908.2.7 33756625

[B51] Luo T Wang SW Xiao N Wang YL Zhou J (2021) A new species of odorous frog genus *Odorrana* (Anura, Ranidae) from southern Guizhou Province, China. Asian Herpetological Research 12(4): 381–398. 10.16373/j.cnki.ahr.200122

[B52] Mahony S (2008) Redescription and generic reallocation of *Ranamawphlangensis* Pillai & Chanda, 1977 (Amphibia: Ranidae). Hamadryad Madras 33(1): 1–12.

[B53] Matsui M (1994) A taxonomic study of the *Rananarina* complex, with descriptions of three new species (Amphibia: Ranidae). Zoological Journal of the Linnean Society 111(4): 385–415. 10.1111/j.1096-3642.1994.tb01489.x

[B54] Matsui M Jaafar I (2006) A new cascade frog of the subgenusOdorrana from peninsular Malaysia. Zoological Science 23(7): 647–651. 10.2108/zsj.23.647 16908965

[B55] Matsui M Tomohiko S Hidetoshi OT Tanaka U (2005) Multiple invasions of the Ryukyu Archipelago by Oriental frogs of the subgenusOdorrana with phylogenetic reassessment of the related subgenera of the genus *Rana*. Molecular Phylogenetics and Evolution 37(3): 733–742. 10.1016/j.ympev.2005.04.030 15964212

[B56] Mo YM Chen WC Wu HY Zhang W Zhou SC (2015) A new species of *Odorrana* inhabiting complete darkness in a karst cave in Guangxi, China. Asian Herpetological Research 6(1): 11–17. 10.16373/j.cnki.ahr.140054

[B57] Orlov NL Natalia B Cuc HT (2006) A new cascade frog (Amphibia: Ranidae) from central Vietnam. Russian Journal of Herpetology 13(2): 155–163.

[B58] Pham CT Nguyen TQ Bernardes M Nguyen TT Ziegler T (2016a) First records of *Bufogargarizans* Cantor, 1842 and *Odorranalipuensis* Mo, Chen, Wu, Zhang et Zhou, 2015 (Anura: Bufonidae, Ranidae) from Vietnam. Russian Journal of Herpetology 23(2): 103–107.

[B59] Pham CT Nguyen TQ Le MD Bonkowski M Ziegler T (2016b) A new species of *Odorrana* (Amphibia: Anura: Ranidae) from Vietnam. Zootaxa 4084(3): 421–435. 10.11646/zootaxa.4084.3.7 27394273

[B60] Pillai RS Chanda SK (1977) Two new species of frogs (Ranidae) from Khasi Hills, India. Journal of the Bombay Natural History Society 74: 136–140.

[B61] Ronquist FR Huelsenbeck JP (2003) MrBayes3: Bayesian phylogenetic inference under mixed models. Bioinformatics 19(12): 1572–1574. 10.1093/bioinformatics/btg180 12912839

[B62] Saikia B Sinha B Kharkongor IJ (2017) *Odorranaarunachalensis*: A new species of Cascade Frog (Anura: Ranidae) from Talle Valley Wildlife Sanctuary, Arunachal Pradesh, India. Journal of Bioresources 4: 30–41.

[B63] Shen HJ Zhu YJ Li Z Chen Z Chen XH (2020) Reevaluation of the holotype of *Odorranaschmackeri* Boettger, 1892 (Amphibia: Anura: Ranidae) and characterization of one cryptic species in *O.schmackeri* sensu lato through integrative approaches. Asian Herpetological Research 11(4): 297–311. 10.16373/j.cnki.ahr.200097

[B64] Simon C Frati F Beckenbach A Crespi B Liu H Flook P (1994) Evolution, weighting andphylogenetic utility of mitochondrial gene sequences and a compilation of conserved polymerase chain reaction primers. Annals of the Entomological Society of America 87(6): 651–701. 10.1093/aesa/87.6.651

[B65] Song HM Zhang SY Qi S Lyu ZT Zeng ZC Zhu YH Huang MH Luan FC Shu ZF Gong Y Liu ZF Wang YY (2023) Redefinition of the *Odorranaversabilis* Group, with a New Species from China (Anura, Ranidae, *Odorrana*). Asian Herpetological Research 14(4): 283–299. 10.3724/ahr.2095-0357.2023.0019

[B66] Stejneger L (1901) Diagnoses of eight new batrachians and reptiles from the Riu Kiu Archipelago, Japan. Proceedings of the Biological Society of Washington 14: 189–191.

[B67] Stuart BL Bain RH (2005) Tree new species of spinule-bearing frogs allied to *Ranamegatympanum* Bain, Lathrop, Murphy, Orlov & Ho, 2003 from Laos and Vietnam. Herpetologica 61(4): 478–492. 10.1655/05-06.1

[B68] Stuart BL Chanard T (2005) Two new *Huia* (Amphibia: Ranidae) from Laos and Thailand. Copeia 2005(2): 279–289. 10.1643/CH-04-137R3

[B69] Stuart BL Chuaynkern Y Chan-ard T Inger RF (2006a) Tree new species of frogs and a new tadpole from eastern Thailand. Fieldiana. Zoology 111: 1–19. 10.3158/0015-0754(2006)187[1:TNSOFA]2.0.CO;2

[B70] Stuart BL Inger RF Voris HK (2006b) High level of cryptic species diversity revealed by sympatric lineages of Southeast Asian forest frogs. Biology Letters 2(3): 470–474. 10.1098/rsbl.2006.0505 17148433 PMC1686201

[B71] Su X Wu XB Yan P Cao SY Hu YL (2007) Rearrangement of a mitochondrial tRNA gene of the concave-eared torrent frog, *Amolopstormotus*. Gene 394(1–2): 25–34. 10.1016/j.gene.2007.01.022 17368759

[B72] Tamura K Stecher G Peterson D Fiipski A Kumar S (2013) MEGA6: Molecular evolutionary genetics analysis, version 6.0. Molecular Biology and Evolution 30(12): 2725–2729. 10.1093/molbev/mst197 24132122 PMC3840312

[B73] Tran TT Orlov NL Nguyen TT (2008) A new species of Cascade frog of *Odorrana* Fei, Yi et Huang, 1990 genus (Amphibia: Anura: Ranidae) from Bac Giang Province (Yen Tu Mountain Range, northeast Vietnam). Russian Journal of Herpetology 15: 212–224.

[B74] Wang YY Lau N Yang JH Chen GL Liu ZY Pang H Liu Y (2015) A new species of the genus *Odorrana* (Amphibia: Ranidae) and the first record of *Odorranabacboensis* from China. Zootaxa 3999(2): 235–254. 10.11646/zootaxa.3999.2.4 26623573

[B75] Werner F (1930) *Ranaleporipes*, a new species of frog from South China, with field notes by R. Mell. Lingnan Science Journal 9: 45–47.

[B76] Wu GF (1977) A new species of frogs from Huang-Shan, Anhui, *Amolopstormotus* Wu. Dong Wu Xue Bao 23: 113–115.

[B77] Wu L Xu RH Dong Q Li DJ Liu JS (1983) A new species of *Rana* and records of amphibians from Guizhou province. Dong Wu Fen Lei Xue Bao 29(1): 66–70.

[B78] Xue R Liu JB Yu JJ Yang JD (2016) The complete mitogenome of *Amolopsloloensis* and related phylogenetic relationship among Ranidae. Mitochondrial DNA. Part A, DNA Mapping, Sequencing, and Analysis 27(6): 4629–4630. 10.3109/19401736.2015.1101589 26681370

[B79] Yang DT (2008) Amphibia and Reptilia of Yunnan. Yunnan Science and Technology Press, Kunming, 65–81.

[B80] Yang DT Li SM (1980) A new species of the genus *Rana* from Yunnan. Zoological Research 1(2): 261–264.

[B81] Ye CY Fei L (2001) Phylogeny of genus *Odorrana* (Amphibian: Ranidae) in China. Dong Wu Fen Lei Xue Bao 47(5): 528–534.

[B82] Yu DN Zhang JY Zheng RQ (2012) The complete mitochondrial genome of *Babinaadenopleura* (Anura: Ranidae). Mitochondrial DNA 23(6): 423–425. 10.3109/19401736.2012.710214 22943433

[B83] Yuan ZY Zhou WW Chen X Poyarkov N Chen HM Liaw NHJ Chou WH Matzke H Iizuka K Min MS Kuzmin S Zhang YP Che J (2016) Spatiotemporal Diversification of the True Frogs (Genus *Rana*): A Historical Framework for a Widely Studied Group of Model Organisms. Systematic Biology 65(5): syw055. 10.1093/sysbio/syw055 27288482

[B84] Zhang B Li Y Hu K Li P Gu Z Xiao N Yang D (2021) A new species of *Odorrana* (Anura, Ranidae) from Hunan Province, China. ZooKeys 1024: 91–115. 10.3897/zookeys.1024.56399 33776522 PMC7985132

[B85] Zhu YJ (2016) Genetic Differentiation of *Odorranaschmackeri* Species Complex. Henan Normal University, Henan.

